# Estimating the number needed to treat from continuous outcomes in randomised controlled trials: methodological challenges and worked example using data from the UK Back Pain Exercise and Manipulation (BEAM) trial

**DOI:** 10.1186/1471-2288-9-35

**Published:** 2009-06-11

**Authors:** Robert Froud, Sandra Eldridge, Ranjit Lall, Martin Underwood

**Affiliations:** 1 Centre for Health Sciences, Barts and the London School of Medicine and Dentistry, London, E1 2AT, UK; 2 Warwick Clinical Trials Unit, Warwick Medical School, Gibbet Hill Road, Coventry, CV4 7AL, UK

## Abstract

**Background:**

Reporting numbers needed to treat (NNT) improves interpretability of trial results. It is unusual that continuous outcomes are converted to numbers of individual responders to treatment (i.e., those who reach a particular threshold of change); and deteriorations prevented are only rarely considered. We consider how numbers needed to treat can be derived from continuous outcomes; illustrated with a worked example showing the methods and challenges.

**Methods:**

We used data from the UK BEAM trial (*n *= 1, 334) of physical treatments for back pain; originally reported as showing, at best, small to moderate benefits. Participants were randomised to receive 'best care' in general practice, the comparator treatment, or one of three manual and/or exercise treatments: 'best care' plus manipulation, exercise, or manipulation followed by exercise. We used established consensus thresholds for improvement in Roland-Morris disability questionnaire scores at three and twelve months to derive NNTs for improvements and for benefits (improvements gained+deteriorations prevented).

**Results:**

At three months, NNT estimates ranged from 5.1 (95% CI 3.4 to 10.7) to 9.0 (5.0 to 45.5) for exercise, 5.0 (3.4 to 9.8) to 5.4 (3.8 to 9.9) for manipulation, and 3.3 (2.5 to 4.9) to 4.8 (3.5 to 7.8) for manipulation followed by exercise. Corresponding between-group mean differences in the Roland-Morris disability questionnaire were 1.6 (0.8 to 2.3), 1.4 (0.6 to 2.1), and 1.9 (1.2 to 2.6) points.

**Conclusion:**

In contrast to small mean differences originally reported, NNTs were small and could be attractive to clinicians, patients, and purchasers. NNTs can aid the interpretation of results of trials using continuous outcomes. Where possible, these should be reported alongside mean differences. Challenges remain in calculating NNTs for some continuous outcomes.

**Trial Registration:**

UK BEAM trial registration: ISRCTN32683578.

## Background

Measurement, and reporting, of clinical outcomes is crucial to interpretation of randomised controlled trials. The clinical importance of some outcomes, such as death, is usually fairly clear. However, the clinical importance of differences found in patient-reported continuous outcomes, used to assess chronic disorders with variable courses, such as low back pain, is often less clear. With ever-larger trials, and meta-analyses of data from multiple trials, we have the statistical power to demonstrate quite small mean differences in these outcome measures that are unlikely to have arisen by chance. However, the interpretation of clinical importance remains problematic. Summary statistics are, through statistical inference, applicable to a population but results from these studies may be less useful if we want to apply them to an individual. For example, a 5 mm Hg change in blood pressure may be important at a population level but of little relevance to an individual. [1] For chronic disorders with variable courses, the importance of small mean differences in continuous primary outcome measures of interest is less clear. In early 2008 there was considerable media interest in the UK in a meta-analysis of Selective Serotonin Reuptake Inhibitors (SSRIs) that was reported as demonstrating that these were not effective for the treatment of mild to moderate depression http://news.bbc.co.uk/2/hi/health/7263494.stm. [2] This paper has been very influential in informing popular opinion about the use of Selective Serotonin Reuptake Inhibitors, but it contrasts with an earlier meta-analysis in which a similarly small standardised effect size was reported (0.31 compared with 0.32) and the authors concluded that these were superior to placebo. [3,4] It has been suggested that the discord between conclusions stemmed from the use of a standardised effect size to judge clinically important change. [4] Standardised effect sizes, calculated as the between-group mean difference divided by the standard deviation at baseline, are one approach to quantifying effect sizes in trials. Conventionally, 0.2 is considered small, 0.5 medium, and 0.8 large. [5] This approach is widely used to define the magnitude of changes in variables that can be readily observed. Although there is generally a close relationship between the standardised effect size and the proportion of participants who benefit from treatment, [6] this may not always be the case. [7]

Thresholds of minimally important change (MIC) are often used to judge the clinical importance of between-group mean differences. However, simply dichotomising group change as clinically important or not, does not tell us how many individuals benefit from a treatment. Guyatt and colleagues, [7] in 1998, demonstrated the usefulness of assessing individual improvement by considering the example of a trial with a mean effect of 0.25 units on a continuous outcome scale, where the MIC for an individual is 0.5 units. This could represent a situation in which the intervention has no effect in 75% of participants, whilst 25% improve by 1.0 unit, implying that on average one in every four participants treated would gain a clinically important change; the number needed to treat (NNT) is four. When only the mean difference is presented, which is half the magnitude of the MIC for an individual, the intervention is likely to be interpreted as ineffective. In contrast, an NNT of four suggests a highly effective treatment.

How outcomes are presented, can have a substantial effect on the interpretation of results. [8] However, many authors still use only one method. Adding an estimate of the NNT to gain, on average, one additional improvement, may aid interpretation of trials with continuous outcomes that are not intuitively understandable to patients, clinicians, and purchasers; few authors do this. Furthermore, for many common disorders, such as back pain, depression, chronic fatigue, etc, it may be just as important to prevent deteriorations as it is to promote improvement; but few authors who report NNT consider this. We aimed to explore practical challenges of using the NNT to report a patient-reported continuous outcome in a way that is clear to end-users and to explore its implications on the interpretation of a previously reported trial. We report a re-analysis of data from the UK Back Pain Exercise and Manipulation (UK BEAM) trial. [9] The largest benefit from any of the treatments in UK BEAM was 1.87 points on the Roland-Morris disability questionnaire (RMDQ), [10] at three months (a standardised mean difference of 0.47). This is smaller than the 2.5 point between-group difference used for the sample size calculation and it has since been argued that, in light of this, the benefits found in UK BEAM were not clinically important. [11]

In this re-analysis we estimated the NNT for one patient to gain a clinically important improvement and for one patient to *receive a benefit*, defined as either an improvement gained or deterioration prevented.

## Methods

The UK BEAM trial is reported in detail elsewhere. [12] Briefly, 1,334 participants with low back pain lasting for more than four weeks were recruited from 181 practices in the Medical Research Council General Practice Research Framework. They were randomised between the following interventions.

*"Best care" in general practice (the "comparator" treatment) *– General practice teams were trained in "active management" and provided patients with The Back Book. [13,14]*Exercise programme *– An initial assessment and up to nine exercise classes led by physiotherapists in community settings. [15]

*Spinal manipulation package *– The UK chiropractic, osteopathic, and physiotherapy professions agreed to use a package of techniques, during eight sessions over 12 weeks. [16]

*Combined treatment *– Participants received six weeks of manipulation followed by six weeks of exercise. Treatments were those given to the manipulation only or exercise only groups.

### Outcome measures

UK BEAM's primary end point was the change in the RMDQ from baseline to follow-up. [10] This 24-item questionnaire measuring disability is one of the most commonly used outcome measures in trials of back pain. Scores range from 0 to 24; higher scores indicate greater disability. A secondary outcome in UK BEAM was the participants' global perception of change indicated on a health transition question, a single item asking participants if they have experienced improvement or deterioration in their low back pain since beginning treatment. [17] It has seven possible responses: 1. completely recovered, 2. much improved, 3. slightly improved, 4. no change, 5. slightly worsened, 6. much worsened, and 7. vastly worsened. Follow-up was at four weeks, three and 12 months by postal questionnaire. Analyses were based on mean differences between intervention groups and the comparator treatment group. There were no differences between groups at four weeks. Statistically significant positive results were observed for all three interventions at three months, and for manipulation and combined treatment at 12 months (Table 1). Our new analyses are intended to aid interpretation of results unlikely to have arisen by chance, not to change conclusions. We have therefore focused on outcomes that were statistically significant in the original analysis.

**Table 1:** Roland-Morris score decrease in the UK BEAM trial

	Net benefit from intervention			
	
Group	at three months	(95% CI)	at 12 months	(95% CI)
Exercise	1.36**	(0.63–2.10)	0.39	(-0.41–1.19)
Manipulation	1.57***	(0.82–2.32)	1.01*	(0.22–1.81)
Combined treatment	1.87***	(1.15–2.60)	1.30**	(0.54–2.07)

Adapted from UK BEAM BMJ 2004;329:1377–81

* Significant at 5% level

** Significant at 1% level

*** Significant at 0.1% level

### Individual improvement

The measurement precision of the outcome of interest is important when judging the threshold for individual change (whether it is deterioration or improvement). [18] Clinicians are familiar with the concept of taking three blood pressure measurement readings to assess whether individuals are over a treatment threshold for hypertension; this limits measurement error due to the instrument's imprecision and within person variation. The measurement error, of any instrument, is inversely proportional to the number of measurements; either repeated measures on an individual or participants measured in the group. The minimal *detectable *change is dependent on measurement error, and thus depends on the number of measurements. Trials can be designed so that the minimal detectable change, is less than the threshold of minimally *important *change ((MIC) i.e., a magnitude of change that may be considered patient-important). [19-21] However, at an individual level, there is evidence that the minimal detectable change on the RMDQ is larger than the MIC. [19,22-24] This leads to difficulty choosing a threshold by which to judge individual improvement; adopting minimal detectable change as a proxy for importance may not lead to meaningful results; too few participants achieve such large changes. One suggestion is that we measure patients on multiple occasions before and after treatment—this is similar to the approach for measuring blood pressure. However, this may be impractical in studies of low back pain, where a questionnaire is used to assess participants' change.

Similar MIC thresholds on the RMDQ have been identified from different populations using receiver operator characteristic (ROC) curves. [19,23-27] In 2008, after reviewing a mix of literature on the instrument's MIC and minimal detectable change, a group of experts agreed five RMDQ points represented an appropriate threshold by which to judge individual improvement. [28] A further challenge, is that the absolute magnitude of MIC on the RMDQ may increase with baseline severity; [22,23,25,29] this could mean that the MIC for more severely affected participants is larger, or it could be wholly or partly, an artifact due to regression to the mean. To account for this, the group suggested a ≥ 30% improvement from baseline as an alternative threshold for judging individual improvement. [28] It is these values that we have used in our analyses.

### Population-specific comparison

To ensure that it was appropriate to apply the consensus threshold of five points (change from baseline), we examined the MIC and the minimal detectable change in the UK BEAM population. We used ROC curves, using the transition question as the external criterion, to estimate MIC. We categorised participants as improved if their response to the transition question was 'completely recovered' or 'much improved' [30] and defined MIC as the cut-point on the RMDQ corresponding to the highest combination of sensitivity and specificity. [31] We estimated minimal detectable change from the within person and residual error of stable (neither improving nor deteriorating) patients' repeated measurements, between baseline and four weeks (see Additional File 1). [20,32] The four week follow-up data were not used in the original BEAM analysis. We estimated minimal detectable change using RMDQ data from those participants who indicated 'no change' on the transition question at four weeks. To further examine the stability of these participants, we tested for a difference in their RMDQ scores, between baseline and four weeks using Student's *t *test.

Guyatt et al [33] suggest that correlations of less than 0.5 between the change in health related quality of life (HRQoL) score and the transition question, provide grounds for doubting the construct validity of the transition question. Criticisms of using transition questions are that the rating is likely to be highly correlated with the follow-up health state, and that respondents may not correctly recall their initial health state (i.e., the baseline score). To ensure that the transition question is measuring change, and not merely reflecting current health states, a correlation between baseline score and the transition question, and follow-up score and the transition question should ideally be present, equal, and opposite. [33] In addition, in a linear regression model with follow-up score entered as the initial explanatory variable, the baseline score should explain a significant proportion of the residual variance in the transition rating. [33] Thus, in order to explore the validity of our transition question, we calculated Pearson's correlation coefficient between baseline and follow-up RMDQ scores, the change in RMDQ score and the transition question, the baseline score and the transition question, and the follow-up scores and the transition question. Also, we constructed linear regression models, in which the transition question was entered as the dependent variable, and the follow-up scores as the explanatory variables. Subsequently, we added the baseline score to the models. Because of the large number of comparisons, we considered a probability less than 0.01 statistically significant. We performed all analyses using STATA version 10.

### Calculating NNT

We calculated the NNT using the RMDQ for all comparisons with a statistically significant difference in the original analysis. We used two methods of calculation; *method one*, improvements gained, and *method two*, benefits gained (improvements gained+deteriorations prevented).

#### Method one-additional improvements gained

We subtracted the proportion of patients who improved in the control group from those who improved in the intervention group (absolute risk reduction). We then inverted this to obtain the NNT, and calculated 95% confidence intervals using Bender's method, which is based on Wilson scores. [34] The conventional method for calculating 95% confidence intervals for NNTs is based on the simple Wald method, which yields confidence intervals that are, in many cases, too narrow. [34] The application of Wilson score method improves the calculation and presentation of the confidence intervals (See Additional File 1). For the RMDQ we estimated improvements gained using both a five-point reduction between baseline and three months score, and a proportional reduction of ≥ 30% in the baseline score.

#### Method two-benefits gained

To incorporate deteriorations prevented, we calculated the difference in the proportion of improvements minus deteriorations in the intervention group and improvements minus deteriorations in the control group. [35] We then inverted the resulting absolute risk reduction to obtain the NNT. We modified Bender's method of calculating 95% confidence intervals for NNT, to incorporate the extra variance terms introduced through considering both improvements and deteriorations (see Additional File 1).

We used the same improvement thresholds as in method one. As there is no consensus on thresholds for deterioration on the RMDQ, we sought to estimate MIC for deterioration using ROC curves; using the transition question as the external criterion. However, the value generated was negative, implying that, on average, those who reported deterioration had an improved RMDQ score; a paradox that indicated the threshold was unsuitable for use. Therefore, we adopted a ≥ five-point deterioration and a ≥ 30% proportional increase in baseline score, as thresholds for deterioration.

## Results

**Figure 1:**
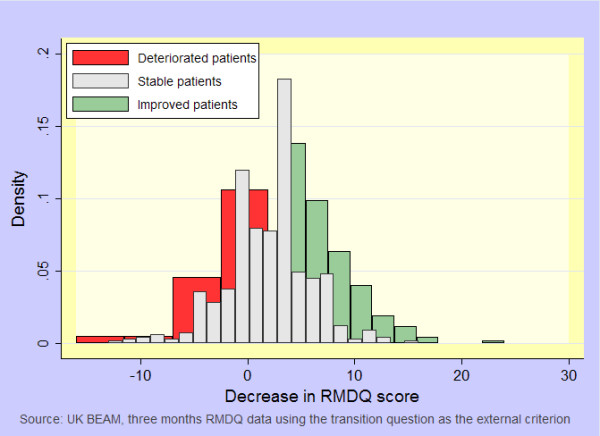
**Score distributions of deteriorating, stable, and improving patients**. Figure one shows the distributions of RMDQ score decrease in patients who were classified as having deteriorated, remaining stable, or having improved on the transition question. One can see that patients who deteriorated (those who reported being 'much worse' or 'vastly worse') have a score change distribution with a mean close to zero (0.4). The MIC cut-off of 4.0 points and the consensus threshold of 5.0 points well separate improved patients from stable patients, in these data. Further research and debate on the MIC cut-off for deterioration is needed.

At three months, complete RMDQ and transition questions were available on 1027/1334 (77%) and 882/1334 (66%) participants respectively. Figure 1 shows the distributions of the RMDQ scores reduction; patents who reported deterioration on the health transition question had a mean decrease of 0.4 RMDQ points. At 12 months, data were available on 994/1334 (75%) and 990/1334 (74%) participants; 640/1334 (48%) participants indicated 'No change' at four weeks. The MIC and minimal detectable change in our population were 4.0 (5.0 using 12 month data) and 8.1 points respectively. Participants who indicated 'No change' at four weeks had a baseline score of 8.5 RMDQ points (*SD *= 3.9) and a four week follow-up score of 6.6 (*SD *= 4.6), *P *< 0.001).

Pearson's correlation coefficient between the baseline and follow-up RMDQ scores was 0.52 (*P *< 0.001) at three months and 0.50 (*P *< 0.001) at one year. The correlation between the change in RMDQ score and the transition question was 0.49 (*P *< 0.001) at three months, and 0.57 (*P *< 0.001) at one year. The correlations between the baseline RMDQ score and the transition question were 0.17 (*P *< 0.001) at three months and 0.22 (*P *< 0.001) at one year. Correlations between the RMDQ follow-up scores and the transition question were 0.57 (*P *< 0.001) at three months, and 0.67 (*P *< 0.001) at one year. The mean RMDQ score at baseline was 9.0 with an SD of 4.0, at three months it was 5.5 with an SD of 5.0, and at one year it was 5.4 with an SD of 5.2. In a linear regression model, the RMDQ follow-up score at three months explained 33% of the variance in transition question rating at three months (*β *= 0.144, *P *< 0.001; the addition of the baseline score to the model was significant and explained an extra 2% of the variance (*β *= -0.056, *P *< 0.001). At one year, the RMDQ follow-up score explained 45% of the variance in the transition question rating (*β *= 0.178, *P *< 0.001); the addition of baseline score to the model was significant and explained a further 2% of the variance (*β *= -0.058, *P *< 0.001).

Table 2 shows the numbers and proportion of participants who improved in each group using either five-points or 30% change as thresholds marking responders to treatment. Methods for calculating 'benefit' and 'improvement' produced similar NNTs using either five-points or 30% change thresholds (Table 3). The ranked effectiveness of the interventions followed the original analysis (Table 1): the largest effect was seen in the combined treatment group, and the smallest in the exercise group.

**Table 2:** Numbers (%) of improved and deteriorated patients

	Improved		Stable		Deteriorated	
*Three months*						
*Best care*						
5 point reduction	62	(24)	181	(70)	13	(5)
30% change	125	(49)	99	(39)	32	(13)
*Exercise*						
5 point reduction	82	(36)	138	(61)	5	(2)
30% change	135	(60)	81	(36)	9	(4)
*Spinal manipulation*						
5 point reduction	125	(44)	148	(52)	14	(5)
30% change	193	(67)	63	(22)	31	(11)
*Exercise and spinal manipulation*						
5 point reduction	117	(45)	135	(52)	7	(3)
30% change	185	(71)	61	(24)	13	(5)
*12 months*						
*Best care*						
5 point reduction	84	(34)	148	(60)	16	(6)
30% change	139	(56)	82	(33)	27	(11)
*Spinal manipulation*						
5 point reduction	125	(46)	133	(49)	15	(5)
30% change	187	(68)	62	(23)	24	(9)
*Exercise and spinal manipulation*						
5 point reduction	115	(45)	133	(52)	9	(4)
30% change	180	(70)	57	(22)	9	(4)

**Table 3:** NNTs derived from consensus thresholds for MIC for the RMDQ (95% CI)

	Exercise		Manipulation		Combined	
*Three months*						
Improvement, 5 points	8.2	(4.9 to 25.0)	5.2	(3.7 to 8.8)	4.8	(3.5 to 7.8)
Improvement, 30%	9.0	(5.0 to 44.5)	5.4	(3.8 to 9.9)	4.4	(3.3 to 7.0)
Benefit, 5 points	6.6	(4.1 to 16.6)	5.1	(3.6 to 9.3)	4.3	(3.1 to 7.0)
Benefit, 30%	5.1	(3.4 to 10.7)	5.0	(3.4 to 9.8)	3.3	(2.5 to 4.9)
*12 months*						
Improvement, 5 points	*	-	8.4	(5.0 to 28.6)	9.0	(5.2 to 37.8)
Improvement, 30%	*	-	8.0	(4.9 to 24.3)	7.1	(4.5 to 17.9)
Benefit, 5 points	*	-	7.8	(4.5 to 29.0)	7.2	(4.3 to 22.8)
Benefit, 30%	*	-	6.9	(4.1 to 21.1)	5.8	(3.7 to 13.2)

* Analyses were not performed as no mean difference was reported between the exercise and best care groups at 12 months

At 12 months, effect sizes were smaller and similar in each group (Table 3).

## Discussion

These new analyses aid interpretation of the trial results. Our analyses illustrate how the practical challenges of incorporating deterioration and allowing for measurement error might be overcome when basing NNTs on patient-reported continuous outcomes. Nevertheless, we were unable to develop a robust threshold for deterioration.

The striking finding here is that, in contrast to the original analysis suggesting at best a small to moderate benefit from the active interventions (Table 1), the NNTs to achieve an improvement/benefit on the RMDQ were small. Even for manipulation at one year, which had the smallest of the statistically significant mean effects, the NNT could be attractive to clinicians, patients, and purchasers. Notably referring only five to six patients for the manipulation package, on average will yield one additional improvement at three months, and, using the most conservative of our estimates, eight to nine referrals, on average will yield additional improvement at one year. There is little difference in NNTs resulting from methods one and two, suggesting that in this case, the active interventions had little effect on preventing or increasing deteriorations.

It is not ideal that our transition question ratings correlate moderately with follow-up scores, and slightly but in the same direction with the baseline score; nevertheless this is not an unusual finding. [33,36] The baseline RMDQ score significantly explained 2% of the residual variance in transition rating in the regression models we fitted. However, this is a trivial proportion. In addition, we found the correlation between the follow-up score and the transition question was greater than the correlation between the change score and the transition question. These findings suggest that participants' health status at the time of follow-up may have been the prime driver of their response to the transition question.

The poor performance of the transition question may have led to inaccurate estimates of MIC and minimal detectable change, as both of these rely upon the transition rating to identify improved or stable patients. However, our estimated MIC value of 4.0 points, falls within the 3.0 to 5.0 range of values reported in other studies using similar methods; [19,22-27] and our minimal detectable change estimate of 8.1 points, falls between the 5.4 to 12.1 range seen in other studies. [19,20,22-24,37] Moreover, both our MIC and minimal detectable change estimates fall within the 2.0 to 8.6 point range considered by the consensus study team. [28] Therefore, notwithstanding the questionable performance of our transition question, we applied the 5 point RMDQ consensus threshold to our population.

Figure 1 shows the distributions of score change for deteriorated, stable and improved patients; it shows that the mean score change in patients who reported deterioration on the health transition question is close to zero. The MIC cut-off point for the highest combination of sensitivity and specificity corresponded to an improvement in RMDQ score, rather than a deterioration as one might expect. This suggests some degree of construct mismatch: participants may have learned to cope with their disability better, even though globally, they felt that their back pain deteriorated. Therefore we adopted the consensus magnitudes we used to define improvement, as proxy magnitudes for deterioration; however, we acknowledge that magnitudes for deterioration may not mimic those for improvement.

Other authors have considered using NNT to report continuous outcome measures. [7,35,38,39] However, the methods propounded either base NNT calculations on group differences, [35,38,39] do not consider measurement error, [7,39] do not consider deteriorations, [38,39] or are not conducive to the derivation of confidence intervals. [7] Calculating NNT from individual improvements, rather than group differences, may more accurately describe the effects of treatment, especially when treatment response is heterogeneous. We have shown that the measurement error can be considered and incorporated into consensus of the change threshold. This threshold is therefore neither MIC, which can be estimated empirically from valid anchors (such as correctly functioning transition questions), nor the minimally detectable change, which can be estimated from a variety of distribution methods (although we favour the method described in Additional File 1[20,32]), but a hybrid of these two properties. A potential weakness of the approach we present to generating this hybrid is its reliance on expert consensus to define the thresholds for individual change. Nevertheless, NNT has been shown to be remarkably robust to small variations in thresholds. [6]

One drawback of using NNT is that statistical power is lost when converting scales to binary outcomes. [7] By virtue of the large sample sizes in UK BEAM, we were generally able to report NNTs with confidence intervals of reasonable widths. Although the simpler Wald method produces confidence intervals that are almost identical to those presented, we prefer confidence intervals derived from Wilson scores; [34] using Wald confidence intervals in studies with smaller sample sizes, or when NNTs are greater than 10 may result in aberrations or be too narrow. [34]

Senn [40] points out, that for continuous outcomes, which vary within persons as well as between persons, an NNT of four may indicate that 25% of patients are likely to benefit whenever the treatment is used or that all patients will benefit 25% of the time. Thus, we cannot isolate individual patients who will benefit using this method; but this does not minimise the usefulness of NNT in aiding decisions about treatment use at a population level.

Wu and Kottke draw attention to other general limitations of NNT. [41] They show that it can be misleading to compare NNTs from different populations, using the example of an intervention for lowering serum cholesterol, which for preventing mortality, has an NNT around 1000 times larger than the NNT for cardiac transplantation. Thus, the intervention for lowering serum cholesterol appears to have a trivial effect compared to cardiac transplantation, and one may be inclined to believe cardiac transplantation to be the more useful technology. However, the first NNT estimate pertains to the entire national population, whereas the second pertains to a population of cardiac transplant candidates. At the level of the entire population, the intervention for lowering serum cholesterol would have an impact on death rates five times greater than cardiac transplantation.

Wu and Kottke also point out that NNT is dependent on time. Consider that at four weeks the proportion of back pain patients improving in treatment group *A *is 20% and in treatment group *B *it is 10%; the relative risk is 0.5, and the NNT is 10. However, at six months if the proportion improving in group *A *is 40% and in group *B *it is 20%; the relative risk is still 0.5, but the NNT becomes five. Comparisons across non-related time points can mislead. We agree with Gorouhi, that it is necessary to specify a time period in order to correctly interpret the NNT. [42]

We used the transition question to help us identify participants who remained stable between baseline and four weeks in an attempt to estimate the population-specific minimal detectable change. This has certain methodological shortcomings. Norman et al, [43] caution against retrospective classification of participants as improved or stable based on a transition question, explaining that it is possible for this to be unrelated to treatment effect. Also, as discussed above, participants' selection of 'No change' may be guided more by their health state at the time, which was subject to within person variation, than by their aggregate change since first measurement. In this study, participants who selected 'No change' on the transition question at four weeks had decreasing RMDQ scores. In light of this, we must consider that our population-specific estimate of minimal detectable change could be inaccurate, and we recommend that in future, this method of retrospectively identifying stable participants is generally avoided.

A number of our analyses were subject to floor and ceiling effects. For example using five points to define an important change, means that a patient with a RMDQ score of four (the lowest score permitted in UK BEAM) could not have reached the improvement threshold and patients with scores of greater than 19 could deteriorate. Similarly, when using the 30% change threshold, although there was no floor effect, participants with scores higher than 18 could not deteriorate. Sensitivity analyses (not presented here) allowing for these effects, produced results similar to our main analyses.

Whilst not wanting to make too much of a post-hoc re-analysis of these data, it is clear that the small NNTs we derived might, if confirmed, make manipulation very attractive to clinicians, patients, and purchasers. This is an important and new observation.

We have demonstrated that patient-reported continuous outcomes can be reported as NNTs; these aid interpretation. US Food and Drug Administration guidance states that when clinical trials show small mean effect sizes it may be more informative to look at individual rather than group responses. [44] It also states that the definition of an individual 'responder' should be based on pre-specified criteria backed by empirically derived evidence. Following consensus on appropriate thresholds of individual change, analysis in the manner we describe is both facilitated and the logical next step. This raises the extremely important question, as to whether reporting results in this way should be the norm in trials assessing disorders with chronic variable courses such as depression, back pain or chronic fatigue. If the same pattern we have shown here was seen in trials of Selective Serotonin Reuptake Inhibitors, then in contrast with the conclusions of Kirsch and colleagues, [2] we might conclude that they were good enough to justify their routine use for mild to moderate depression.

Future agreement on thresholds for deterioration would permit the estimation for NNT for benefits gained and a more comprehensive picture of the effect of treatment could be portrayed. In some instances, especially where desirable correlations can be established between the HRQoL measure and the transition question, the transition question may be useful and aid interpretation of outcomes.

Finally, it is not our intention to suggest reporting continuous outcomes using NNT should replace conventional analysis, which is necessary to ensure between-group differences are statistically significant rather than chance occurrences, and which preserves statistical power. These analyses are complementary and aid clinical interpretation. [7]

## Conclusion

In contrast to the small mean differences originally reported, NNTs were small and could be attractive to clinicians, patients and purchasers. How results of clinical trials are presented could have important implications for how they are interpreted, and how their findings are implemented. Reporting outcomes of clinical trials using mean differences may not give a full picture of the effect of treatments on patient health, especially when the response to treatment is heterogeneous. Reporting the NNT is currently challenging due to difficulties in defining thresholds of individual improvement that encompass both within patient variation/measurement error and clinically important change. Where possible, trialists should consider reporting NNTs alongside mean differences to aid interpretation.

## Competing interests

RF is a practising osteopath. MU is the Chair of NICE low back pain Guideline Development Group and was a member of the UK BEAM study team.

## Authors' contributions

RF participated in the conception of this new analysis, modification of the Wilson score method used by Bender (Newcombe's method 10), analysis of data, wrote the first draft, and contributed to its critical review. SE contributed to the design and analysis of the study, modification of the Wilson score method used by Bender (Newcombe's method 10), interpretation of results, and commented in detail on successive drafts of the paper. RL commented on the statistical analysis in the paper. MU generated funding for RF's studentship, participated in the conception of this new analysis, interpretation of the analyses, and has commented in detail on successive drafts of the paper. All authors read and approved the final manuscript.

## Pre-publication history

The pre-publication history for this paper can be accessed here:

http://www.biomedcentral.com/1471-2288/9/35/prepub

## Supplementary Material

Additional file 1 **Supplement**. A word document detailing equations for both of the methods described. Stata modules to perform these tasks are available from the corresponding author on request. Click here for file
