# Supplementary material for: Estimating the number needed to treat from continuous outcomes in randomised controlled trials: methodological challenges and worked example using data from the UK Back Pain Exercise and Manipulation (BEAM) trial
Source: BMC Med Res Methodol. 2009 Jun 11;9:35. doi: 10.1186/1471-2288-9-35 (PMC2702335; doi:10.1186/1471-2288-9-35)
Supplement: Additional file 1 — Supplement. A word document detailing equations for both of the methods described. Stata modules to perform these tasks are available from the corresponding author on request. [file 1471-2288-9-35-S1.doc]

**Supplement**

We calculated NNT and confidence intervals for ‘improvement’ (Method one) as follows. [34]

Let *n*_0_ and *n*_1_ be the number of patients in the control and the treatment group, respectively, and let *e*_0_ and *e*_1_ be the number of patients improving in the control and the treatment group, respectively. The probability of ‘improvement’ in the two groups can then be estimated by the proportions *p*_0_ = *e*_0_/*n*_0_ and *p*_1_ = *e*_1_/*n*_1_. The effect measures can be estimated by ARR = *p*_0_ − *p*_1_ and NNT = 1/(*p*_0_ − *p*_1_). Using this notation, the 100 × *(*1 − *α)*% confidence interval for ARR based upon Wilson scores is given by:


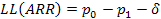


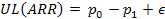


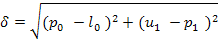


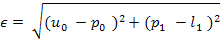


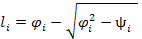


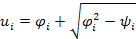


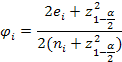


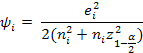


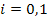


Where z_1-a/2_ is the (1-a/2)-quantile of the standard normal distribution.

The corresponding confidence limits for NNT can then be calculated by LL(NNT)=1/UL(ARR) and UL(NNT)=1/LL(ARR).

In order to calculate NNT and confidence intervals for ‘benefit’ (Method two), we modified the Wilson score method, as described by Bender, [34] by adding variance terms for deterioration. This was done as follows.

Let *e_imp_*_0_ and *e_imp_*_1_ be the number of patients improving in the control and the treatment group, respectively, and let *e_det_*_0_ and *e_det_*_1_ be the number of patients deteriorating in the control and the treatment group, respectively. The probability of ‘improvement’ in the two groups can then be estimated by the proportions *p*_imp0_ = *e*_imp0_/*n*_0_ and *p_imp_*_1_ = *e_imp_*_1_/*n*_1_. The probability of ‘deterioration’ in the two groups can then be estimated by the proportions *p*_det0_ = *e*_det0_/*n*_0_ and *p_det_*_1_ = *e_det_*_1_/*n*_1_. The effect measures can be estimated by ARR = *p*_imp0_ – *p*_det0_ – *p*_imp1_ + *p*_det1_ and NNT = 1/( *p*_imp0_ – *p*_det0_ – *p*_imp1_ + *p*_det1_). Using this notation, the 100 × *(*1 − *α)*% confidence interval for ARR based upon Wilson scores is given by:


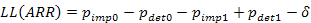


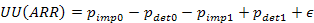


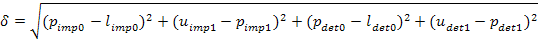


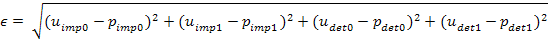


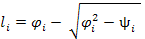


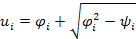


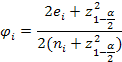


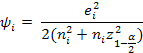


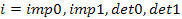


The corresponding confidence limits for NNT can then be calculated by LL(NNT)=1/UL(ARR) and UL(NNT)=1/LL(ARR).

We calculated minimally detectable change as follows.


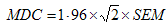


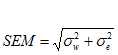


Where *MDC* is the minimally detectable change, *SEM* is the standard error of measurement, *σ_w_^2^* is the within person variance of stable participants’ repeated measurements, and *σ_e_^2^=*the residual variance of stable participants’ repeated measurements.
